# Dasatinib promotes muscle differentiation and disrupts normal muscle regeneration

**DOI:** 10.7150/ijms.94938

**Published:** 2024-05-27

**Authors:** Nanami Ishida, Tamaki Kurosawa, Momo Goto, Noriyuki Kaji, Yayoi Tokunaga, Taiki Mihara, Masatoshi Hori

**Affiliations:** 1 Laboratory of Veterinary Pharmacology, Department of Veterinary Medical Sciences, Graduate School of Agriculture and Life Sciences, Tokyo University, 1-1-1 Yayoi, Bunkyo-ku, Tokyo, 113-8657, Japan.; 2 Laboratory of Veterinary Pharmacology, School of Veterinary Medicine, Azabu University, 1-17-71, Fuchinobe, Chuo-ku, Sagamihara, Kanagawa, 252-5201, Japan.

**Keywords:** Dasatinib, muscle regeneration, C2C12, CTX injury model

## Abstract

Dasatinib is one of the second-generation tyrosine kinase inhibitors used to treat chronic myeloid leukemia and has a broad target spectrum, including KIT, PDGFR, and SRC family kinases. Due to its broad drug spectrum, dasatinib has been reported at the basic research level to improve athletic performance by eliminating senescent cell removal and to have an effect on muscle diseases such as Duchenne muscular dystrophy, but its effect on myoblasts has not been investigated. In this study, we evaluated the effects of dasatinib on skeletal muscle both under normal conditions and in the regenerating state. Dasatinib suppressed the proliferation and promoted the fusion of C2C12 myoblasts. During muscle regeneration, dasatinib increased the gene expressions of myogenic-related genes (*Myod*, *Myog*, and *Mymx*), and caused abnormally thin muscle fibers on the CTX-induced muscle injury mouse model. From these results, dasatinib changes the closely regulated gene expression pattern of myogenic regulatory factors during muscle differentiation and disrupts normal muscle regeneration. Our data suggest that when using dasatinib, its effects on skeletal muscle should be considered, particularly at regenerating stages.

## Introduction

Dasatinib is a second-generation tyrosine kinase inhibitor effective against leukemia and several types of cancer 1. It is approximately 300 times more potent for ABL kinase inhibition than imatinib, a first-generation tyrosine kinase inhibitor *in vitro* 2. It has a broad target spectrum, including KIT, platelet-derived growth factor receptor (PDGFR), and SRC family kinases, and affects imatinib-resistant chronic myeloid leukemias 3. Because of its broad efficacy, dasatinib is also being considered for use in diseases other than cancer at the level of basic research. A cocktail treatment of dasatinib and quercetin has been used as a senolytic drug to remove senescent cells in several organs 4-7. It improved athletic performance in progeroid Ercc1-/- mice and recovered age-related loss of muscle regeneration 8-10. Dasatinib has also been reported as a potential therapeutic drug for Duchenne muscular dystrophy (DMD) to block damaging signaling by phosphorylation and degradation of β-dystroglycan via Src tyrosine kinase inhibition 11, 12. Thus, some data indicate the positive effects of dasatinib on skeletal muscle disease, but its effects on muscle tissue under normal and regenerating conditions have not been investigated.

Skeletal muscle tissue accounts for approximately 30% of body weight and is responsible for our physical movement. Muscle tissues consist of two major contents: bundles of multinucleated myofibers and a series of interstitial connective tissues in those gaps that attach muscle to bone. Muscle tissues have a high regenerative capacity even in the adult state, and satellite cells have a crucial role in muscle regeneration as muscle stem cells. Satellite cells usually stay in quiescence under the muscle membrane. After muscle injury, they are activated and proliferate immediately, undergoing a process of differentiation and fusion that generates multinucleated myofibers 13. During the regeneration, mesenchymal stromal progenitors (MSPs) that construct the muscle connective tissues also proliferate in the muscle interstitium and support myoblast regeneration 14. It is complicated to identify the myogenesis regulatory factors and cell-cell fusion regulatory factors separately, as cell fusion occurs following the progression of myoblast differentiation. One of the few reports on pathways regulating myoblast fusion is that the ERK- retinoid X receptor (RXR) signaling pathway regulates the process by which mononuclear myoblasts fuse with myofibers 15. Nilotinib, classified as a second-generation tyrosine kinase inhibitor, the same as dasatinib, has been reported to inhibit myogenesis by blocking p38 and activating the ERK pathways 16. Dasatinib has been reported to inhibit ERK 17, but its effect on muscle regeneration and fusion has not been investigated.

In this study, we investigated the effects of dasatinib on the skeletal muscle under normal and regenerating conditions. Using C2C12 myoblasts and a cardiotoxin (CTX)-induced muscle injury mouse model, we determined that dasatinib upregulates the expression of myogenesis-related genes during muscle differentiation and disrupts normal muscle regeneration.

## Results

### Dasatinib did not cause myofiber degeneration or necrosis in normal muscle tissue

**Figure 1:**
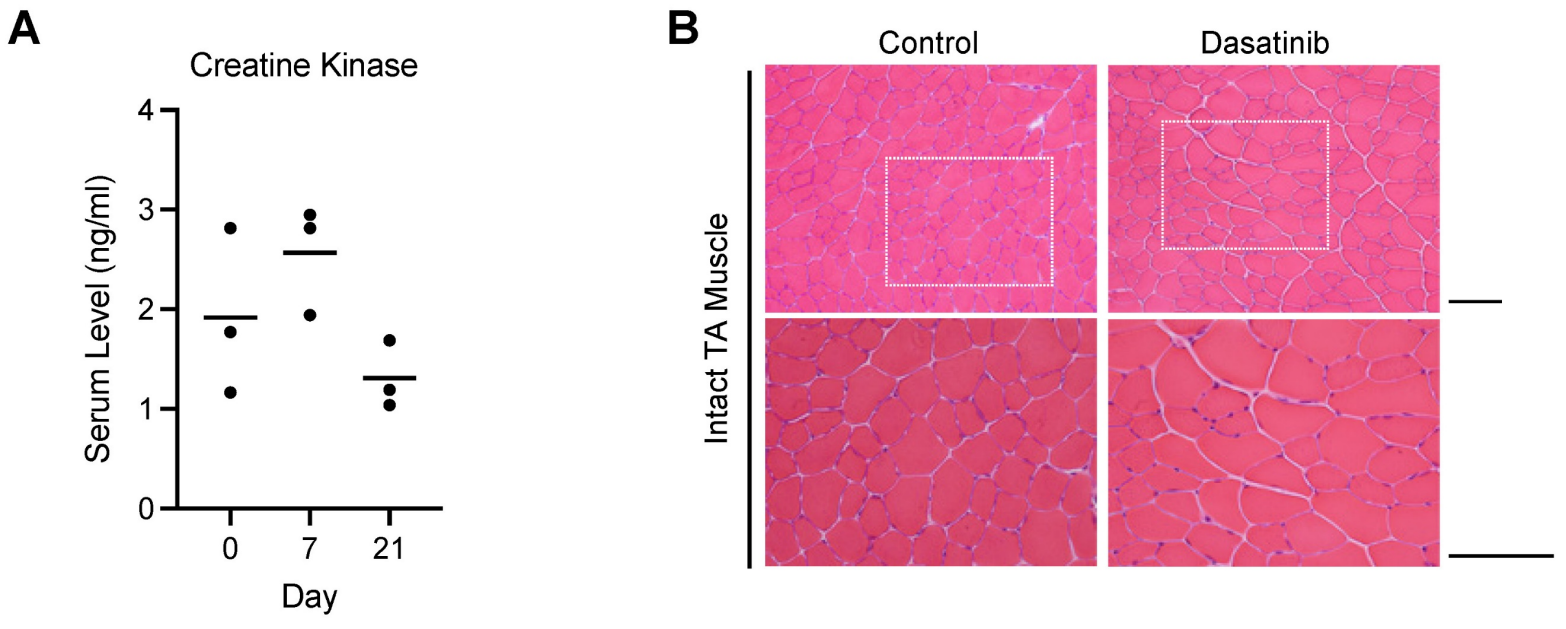
** Dasatinib does not course severe muscle tissue damages in normal conditions. A** Serum levels of creatine kinase at day 0, 7, and 21 with dasatinib treated mice were measured using ELISA. *n* = 3 mice were analyzed at each time point. **B** Representative images of H&E staining of TA muscle cross-sections at 21 days without or with dasatinib treatment are shown. Scale bar = 100 μm.

Since increased serum creatinine kinase levels and rhabdomyolysis have been reported as a side effect of dasatinib in clinical cases 18-20, we first investigated the effect of dasatinib on normal muscle tissues. Dasatinib did not increase the creatine kinase levels in the blood serum at 7 and 21 days after daily intraperitoneal administration of 10 mg/kg/day dasatinib (Fig. 1A). Hematoxylin and eosin (H&E) staining of the tibialis anterior (TA) muscle of mice showed that even treated with dasatinib for 21 days, dasatinib did not cause morphological abnormalities such as degeneration or necrosis of the muscle fibers (Fig. 1B). These data indicate that dasatinib does not cause severe muscle tissue damages in normal conditions.

### Dasatinib inhibits the proliferation of C2C12 myoblasts and promotes their differentiation and fusion

**Figure 2:**
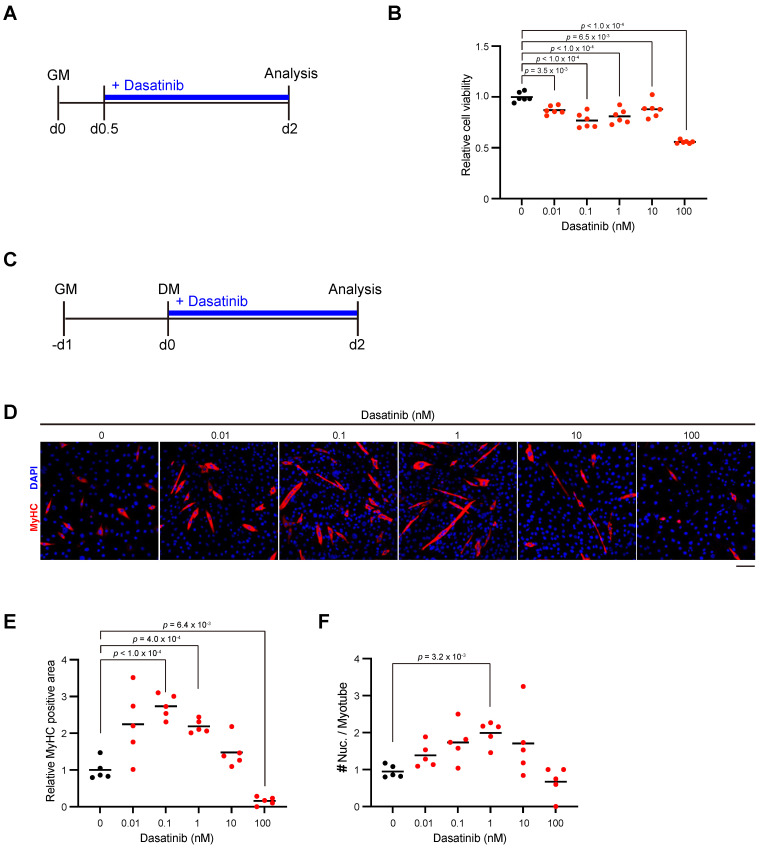
** Dasatinib inhibits C2C12 myoblasts proliferation and promotes their differentiation and fusion. A** Experimental scheme for cell viability test. C2C12 myoblasts were seeded and incubated for 12 hours, followed with dasatinib treatment (0, 0.01, 0.1, 1, 10, and 100 nM) for 36 h. **B** Quantification of cell viability of C2C12 myoblasts treated with dasatinib. **C** Experimental scheme for cell differentiation test. C2C12 myoblasts were seeded and incubated for 24 hours in GM, followed with dasatinib treatment (0, 0.01, 0.1, 1, 10, and 100 nM) for 48 h in DM. **D** Representative images of the C2C12 myoblasts treated with dasatinib (0, 0.01, 0.1, 1, 10, and 100 nM) for 48 h in DM are shown. The myotubes were stained with MyHC (red) and DAPI (blue). Scale bar = 100 μm. **E** MyHC-positive areas were quantified.** F** The nuclear number per myotube was quantified. GM, growth medium; DM, differentiation medium. Data represent individual data points and means. *n*= 5 and 6 independent wells were analyzed for each concentration for (B) and (D-F), respectively. Data were analyzed using a one-way ANOVA or Brown-Forsythe and Welch's ANOVA tests.

We next examined the effect of dasatinib on the proliferation and differentiation of myoblasts. Since nilotinib, which is classified as a second-generation tyrosine kinase inhibitor same with dasatinib, inhibits myogenic differentiation by increasing the proliferation of C2C12 myoblasts 16, we added dasatinib to C2C12 myoblasts during their proliferation state (Fig. 2A). Contrary to the effect of nilotinib, more than a concentration of 0.01 nM dasatinib significantly inhibited the proliferation of C2C12 myoblasts (Fig. 2B). Next, dasatinib was added to C2C12 myoblasts during the early stage of myoblast differentiation (Fig. 2C). Dasatinib increased the myosin heavy chain (MyHC)-positive area at the concentration of 0.1 and 1 nM (Fig. 2D, E). It increased the number of nuclei per myotube at 1 nM (Fig. 2D, F). These data demonstrated that dasatinib inhibited the proliferation of C2C12 myoblasts and promoted their differentiation and cell fusion.

### Dasatinib produces abnormally thin regenerated myofibers and disrupts normal muscle regeneration

**Figure 3:**
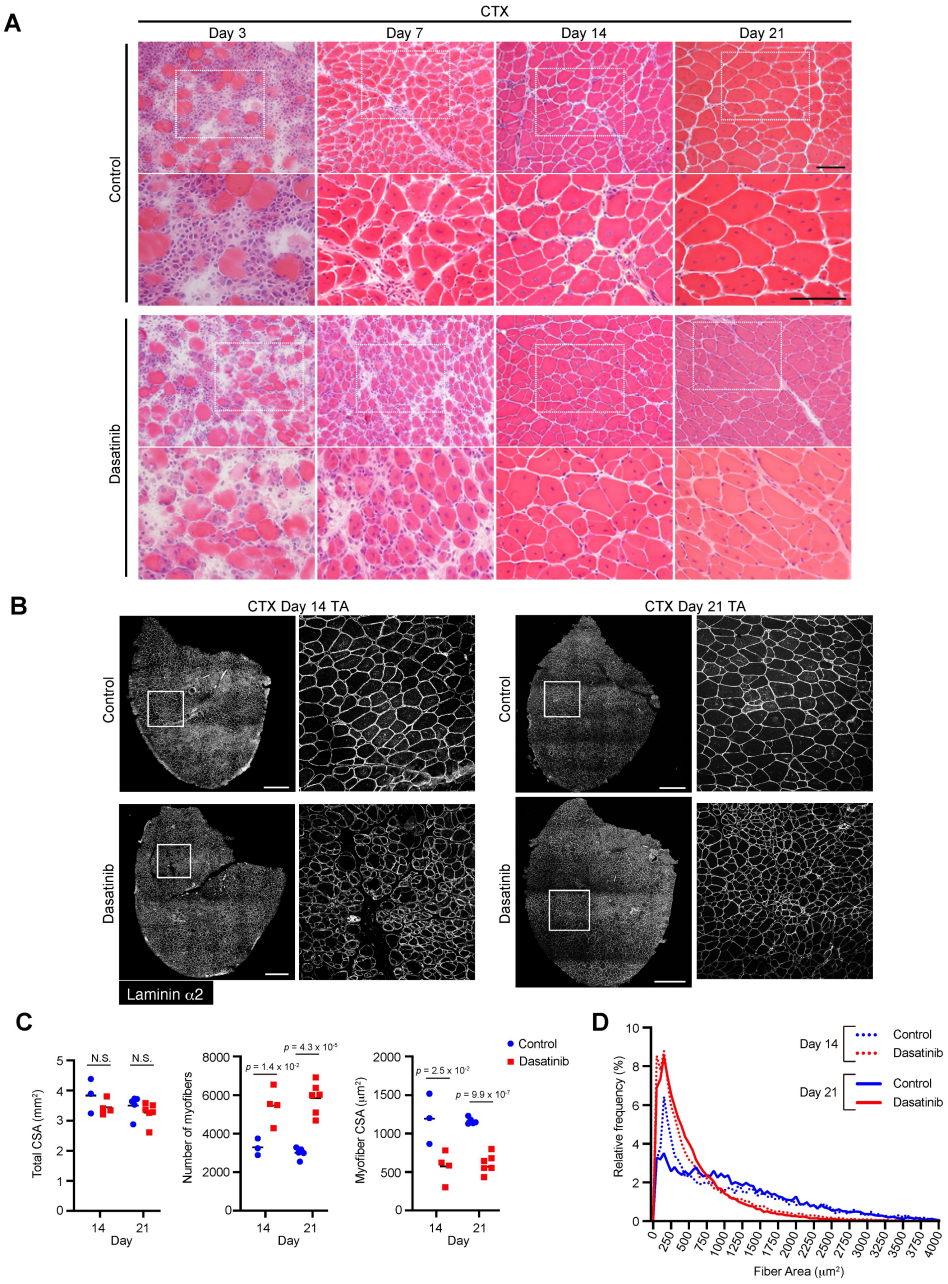
** Dasatinib disrupts normal muscle regeneration and produces thin abnormal muscle fibers. A** Representative images of H&E staining of TA muscle cross-sections from each group at days 3, 7, 14, and 21 post-CTX injury are shown. Scale bar = 100 μm. **B** Representative images of TA cross-sections at days 14 and 21 post-CTX injury stained for laminin α2 are shown. The right panels show the magnified views of the boxed regions on the left panels. Scale bar = 500 μm. **C** Total cross-sectional area (CSA), myofiber CSA, and the number of myofibers in TA muscle at days 14 and 21 post-CTX injury were quantified. *n* = 3 and 6 TA for the control and *n* = 4 and 6 TA treated with dasatinib were analyzed on days 14 and 21 post-CTX injury, respectively. **D** Myofiber CSA distributions in the TA muscle are shown as histograms (lower). *n* = 3 and 6 TA for the control and *n* = 4 and 6 TA treated with dasatinib were analyzed at days 14 and 21 post-CTX injury, respectively. Data represent individual data points and means. Data were analyzed using a two-sided unpaired t-test.

Since dasatinib promoted C2C12 myoblast differentiation and fusion *in vitro*, we examined whether dasatinib affected morphological changes in the regenerated muscles *in vivo*. Dasatinib was injected into CTX-induced muscle-injured mice to evaluate its effects on muscle regeneration. H&E staining revealed that dasatinib induced abnormal thin regenerated myofibers at 14 and 21 days post-CTX injury (Fig. 3A). Immunostaining and historical analysis revealed that the total cross-sectional area (CSA) of TA muscles did not change (Fig. 3B, C), while the number of myofibers increased and the CSA of the myofibers decreased with dasatinib treatment (Fig. 3C, D). Despite nilotinib inhibiting the proliferation of MSPs during muscle regeneration 21, dasatinib did not alter the number of MSPs during muscle regeneration at CTX day 4 (Supplemental Fig. 1). These results indicate that dasatinib produces abnormally thin regenerated myofibers and disrupts normal muscle regeneration.

### Dasatinib upregulates the expression of myogenesis-related genes

**Figure 4:**
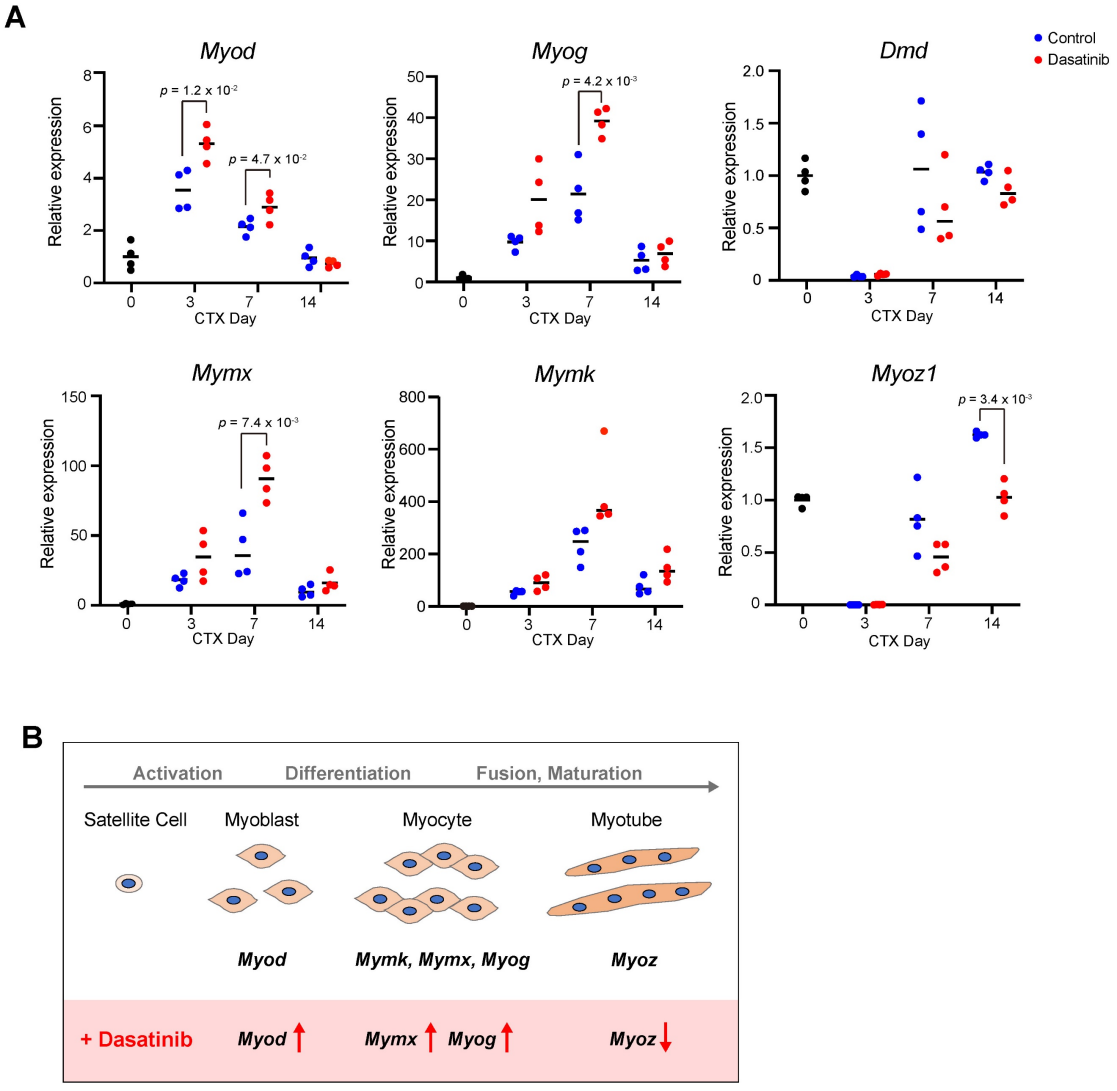
** Dasatinib upregulates the expression of myogenesis-related genes. A** The expression levels of myogenic differentiation genes (*Myod* and *Myog*), muscle-specific fusogenic genes (*Mymk* and* Mymx*), and mature myofiber genes (*Myoz1* and *Dmd*) in TA muscles from WT mice were quantified at days 0, 3, 7, and 14 post-CTX injury. *n* = 4 TA at each time point were analyzed. Data represent individual data points and means. Data were analyzed using a two-sided unpaired t-test and Welch's t-test. **B** Effect of dasatinib on the expressions of myogenic-related genes.

To investigate the mechanism of how dasatinib induces the abnormal muscle regeneration, we analyzed the expression levels of myogenesis-related genes during muscle regeneration with dasatinib treatment. Dasatinib significantly upregulated the expression of *Myod* at 3 and 7 days and *Myog* at 7 days post-CTX injury, respectively (Fig. 4A). In addition, dasatinib significantly upregulated the expression of *Mymx*, a muscle fusion-related gene 22, and tended to upregulate the expression of *Mymk*, another cell fusion-associated gene 23, at 7 days post-CTX injury (Fig. 4A). In contrast, *Myoz1*, a marker of myofiber maturation 24, was significantly downregulated by dasatinib treatment at 14 days post-CTX injury (Fig. 4A). The gene expression level of dystrophin, a component of the basement membrane, remained unchanged (Fig. 4A). Taken together, these data indicate that dasatinib upregulates the expression of several myogenic-related genes but downregulates the expression of myotube maturated gene during muscle regeneration (Fig. 4B).

### Dasatinib disrupts the distribution pattern of fiber type in regenerated muscle

As *Myoz1*, a marker of myofiber maturation, was downregulated by dasatinib treatment, we further examined the regenerated myofibers.

**Figure 5:**
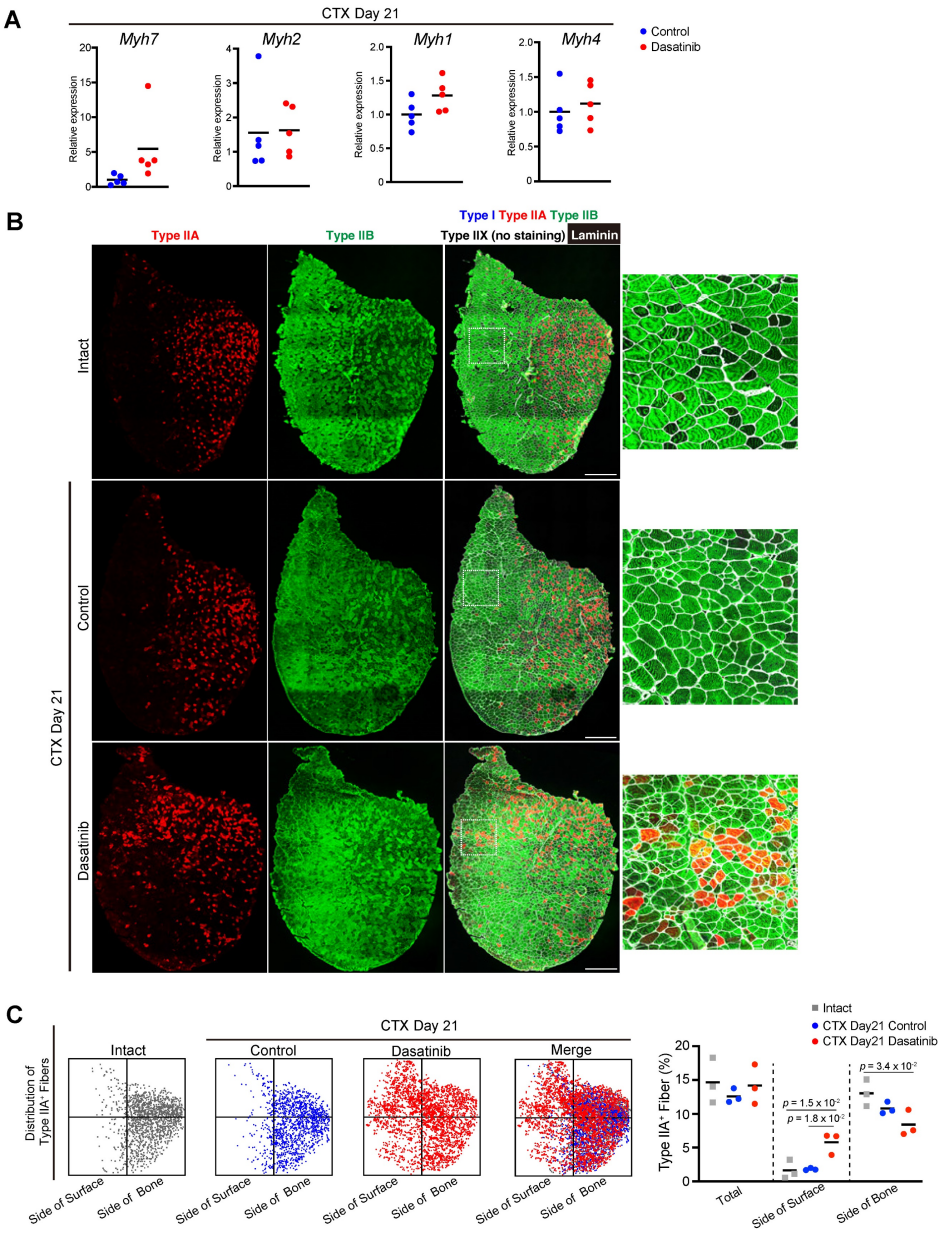
** Dasatinib disrupts the distribution pattern of fiber type in regenerated muscle. A** The expression levels of *Myh7* (type I), *Myh2* (type IIA), *Myh1* (type IIX), and *Myh4* (type IIB) are shown. Data represent individual data points and means. *n* = 5 TA at each time point were analyzed. **B** Representative images of immunohistochemical staining for tibialis anterior (TA) muscle sections from mice with or without dasatinib treatment. Myofibers were stained for MyHC I (blue), MyHC IIA (red), MyHC IIB (green), and laminin (white). The right panels show the magnified views of the boxed regions in the left panels. Scale bar = 500 μm. **C** Distribution pattern of type IIA-positive fibers. Each dot corresponds to a type IIA-positive myofiber (left). The percentage of type IIA-positive fibers in each area is shown in the graph (right). *n* = 3 TA for each group were analyzed. Data were analyzed using a two-sided unpaired t-test or a one-way ANOVA test.

Myofibers that compose muscle tissue are classified into four types based on a particular MyHC isoform: MyHC I, IIA, IIX, and IIB encoded by *Myh7*, *Myh2*, *Myh1,* and *Myh4,* respectively 25. At 21 days post-CTX injury, there was no difference in the expression levels of *Myh* genes between the control and dasatinib-treated groups (Fig. 5A). We also checked the patterning of fiber types of regenerated muscles. Generally, type IIA fibers are distributed on the deep side of the body near the bone in rodents 26, and this distribution pattern is reproduced after muscle regeneration. Immunostaining of the fiber types revealed that dasatinib disrupted the distribution pattern of the type IIA fibers after muscle regeneration (Fig. 5B). Dasatinib altered the distribution of the type IIA fibers closer to the surface than in control (Fig. 5B, C). Dasatinib did not alter the ratio of type IIA muscle fibers (Fig. 5C). These findings demonstrate that dasatinib did not prevent the expression levels of *Myh* genes and did not change the present rate of type IIA fibers but disrupted the normal fiber distribution in regenerated muscles.

### Dasatinib may upregulate the expression of myogenesis-related genes via RXR signaling pathways

**Figure 6:**
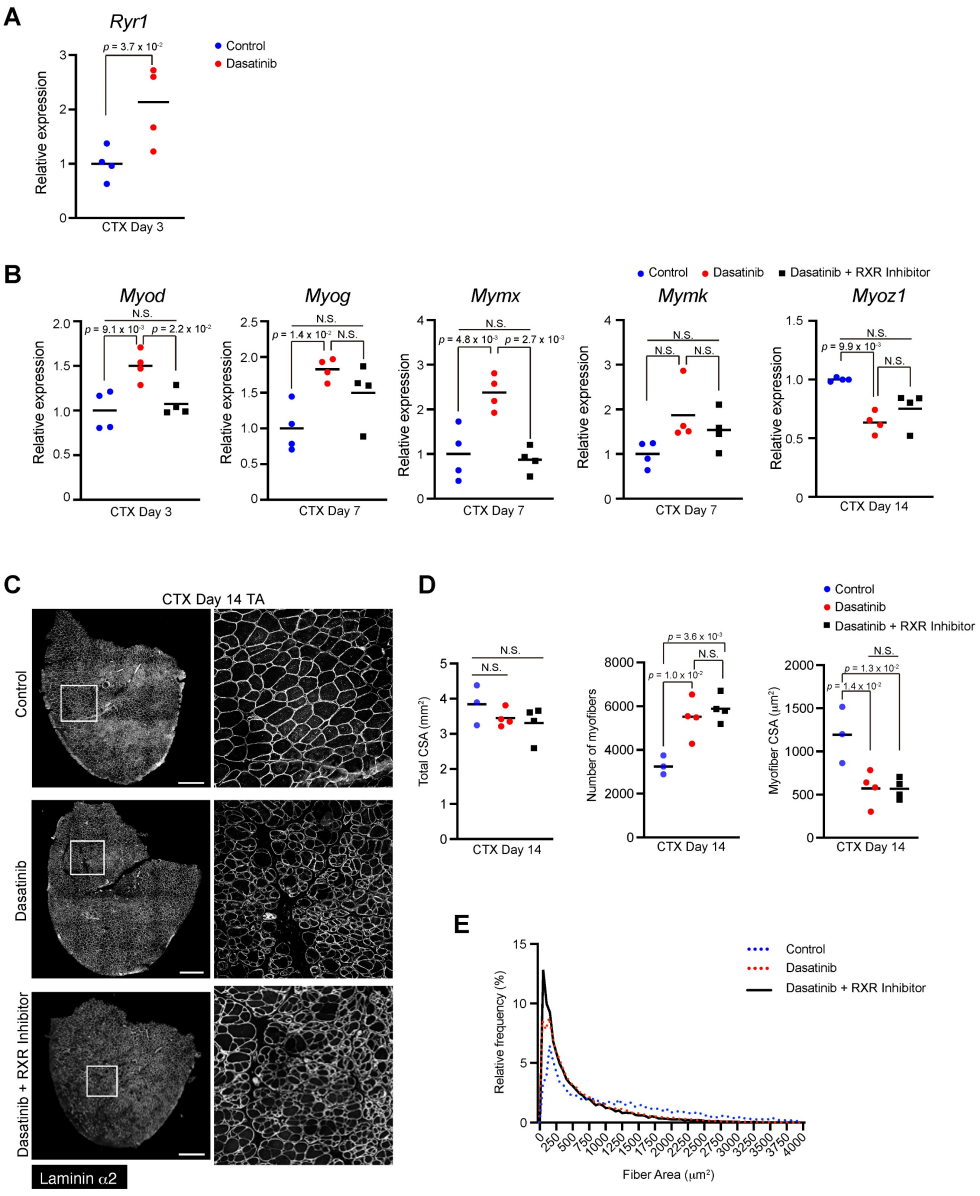
** Dasatinib may upregulate the expression of myogenesis-related genes via RXR signaling pathways. A** The expression level of *Ryr1* gene in TA muscles from WT mice at day 3 post-CTX injury was quantified.** B** Expression levels of myogenic differentiation genes (*Myod* and *Myog*), muscle-specific fusogenic genes (*Mymk* and* Mymx*), and mature myofiber genes (*Myoz1*) in TA muscles from WT mice at days 3, 7, and 14 post-CTX injury were quantified. *n* = 4 TA at each time point. Data represent individual data points and means. Data were analyzed using a two-sided unpaired t-test or a one-way ANOVA test.** C** Representative images of TA cross sections at day 14 post-CTX injury stained for laminin α2 are shown. The right panels show magnified views of the boxed regions on the left panels. Scale bar = 500 μm. **D** Total cross-sectional area (CSA), myofiber CSA and the number of myofibers in TA muscle at day 14 post-CTX injury were quantified. *n* = 3 TA for control, *n* = 4 TA for treated with dasatinib, and *n* = 4 TA for treated with dasatinib with RXR inihibitor were analyzed at days 14 post-CTX injury, respectively. **E** Myofiber CSA distributions in TA muscle are shown as a histogram. *n* = 3 TA for control, *n* = 4 TA for treated with dasatinib, and *n* = 4 TA for treated with dasatinib with RXR inihibitor were analyzed at days 14 post-CTX injury, respectively. Data represent individual data points and the means. Data were analyzed using the one-way ANOVA test.

While dasatinib targets various target kinases 1-3, we focused on the ERK- RXR signaling pathway involved in myogenesis and cell fusion 15. Because ERK regulates muscle differentiation by suppressing RXR activation, and dasatinib has been reported to inhibit ERK 17, we hypothesized that dasatinib might affect muscle differentiation by regulating the ERK-RXR signaling pathway. As expected, dasatinib upregulated the gene expression of *Ryr1*, which is downstream of RXR, at 3 days post-CTX injury (Fig. 6A). Treatment with an RXR inhibitor restored the expression of some myogenesis- and cell fusion-related genes that were upregulated by dasatinib (Fig. 6B). However, the morphological changes in the regenerated muscles induced by dasatinib were not mitigated even with the RXR inhibitor (Fig. 6C-E). This finding suggests that dasatinib may induce abnormal muscle regeneration via RXR signaling, but other pathways are also involved in producing abnormally thin regenerated myofibers.

## Discussion

In this study, we showed that dasatinib does not cause severe muscle tissue damage in normal conditions, but under a regenerating state, it causes abnormally thin regenerated myofibers and disrupts normal muscle regeneration.

Since MSPs have platelet-derived growth factor receptor α (PDGFRα) and are the origin of adipogenesis and fibrosis in skeletal muscle under pathological conditions, several tyrosine kinase inhibitors have been investigated as potential candidates to suppress fibroblast proliferation in DMD. For example, imatinib, a first-generation tyrosine kinase inhibitor, inhibited the proliferation of MSPs and reduced the fibrosis of the DBA/2-mdx, a muscular dystrophy mice model 27. Nilotinib, classified as a second-generation tyrosine kinase inhibitor, the same as dasatinib, inhibits the proliferation of MSPs and reduces extracellular matrix deposition during muscle regeneration 21. On the other hand, dasatinib did not inhibit the proliferation of MSPs (Supplemental Fig. 1), possibly due to its lower selectivity for PDGFR than other tyrosine kinase inhibitors 28. The effects of tyrosine kinase inhibitors on myoblasts have also been evaluated, as they must not have a negative effect on myoblasts as a treatment for muscle diseases. Imatinib inhibited the proliferation of primary myoblasts at high concentration treatments above 10 µM 27. Nilotinib promoted myoblast proliferation and inhibited myogenesis by inhibiting p38 and activating the ERK and AKT pathways 16. We found that dasatinib suppressed the proliferation of C2C12 myoblasts (Fig. 2), upregulated the expression of myogenic-related genes (Fig. 4), and caused abnormally thin muscle fibers (Fig. 3). RXR inhibitors could cancel the elevation of some abnormal myogenesis-related gene expressions caused by dasatinib (Fig. 6). However, the RXR inhibitor did not wholly mitigate dasatinib-induced abnormalities in muscle regeneration, suggesting that pathways other than the RXR signaling pathway are also involved in this dysregulation. Because tyrosine kinase inhibitors affect multiple kinase pathways, it is difficult to determine which is involved in abnormal muscle differentiation. In addition, it has been reported that the function of muscle tissue component cells other than myoblasts is essential during muscle regeneration. Macrophage is critical to clear the necrotic myofibers for proper myoblast fusion and form myofibers 29. The importance of motor nerve innervation during myofiber regeneration after CTX-induced muscle injury has also been reported 30. The possibility that dasatinib affects muscle tissue component cells other than myoblasts should be investigated. Further examining the differences between dasatinib and other kinase inhibitors, such as nilotinib and imatinib, which are also tyrosine kinase inhibitors with different active spectra, may help to understand the regulated mechanism for signaling pathways essential for muscle regeneration.

Previous studies have reported that dasatinib and quercetin cocktail therapy improved muscle regeneration in aged mice but impaired it in young mice 31. Young mice showed loss of the cross-sectional area of regenerating muscle fibers after the cocktail therapy, and that is consistent with our data showing that dasatinib caused thin regenerating muscle fibers (Fig. 3). Satellite cells lose their regenerative potential with aging, and muscle regeneration is impaired 32-34. Dasatinib might evoke satellite cells in aged mice and rescue age-related loss of muscle regeneration. In combination with our data, dasatinib could potentially be used to promote muscle regeneration, but the regenerated muscle tissue may differ from the normal one with its treatment. Its use in muscle should, therefore, be further explored. The use of dasatinib in the treatment of cancer may also require prior assessment of the patient's skeletal muscle condition, considering its side effects on the muscle regeneration process.

## Conclusion

During muscle regeneration, dasatinib upregulated the expressions of myogenic-related genes (*Myod*, *Myog*, and *Mymx*), caused abnormally thin muscle fibers, and disrupts normal muscle regeneration.

## Materials and Methods

### Mice

C57BL/6N and *Pdgfra^EGFP^* mice were purchased from Japan SLC (Shizuoka, Japan) and Jackson Laboratory (Bar Harbor, ME, USA; Stock #007669), respectively. The mice were supplied with food and water *ad libitum* and maintained under constant temperature and humidity in a 12-h dark/light cycle. All the animal experiments were approved by the Experimental Animal Care and Use Committee of the University of Tokyo.

### CTX-induced muscle regeneration model

Twelve to fourteen-week-old male C57BL/6N and *Pdgfra^EGFP^* mice were randomly divided into sunflower oil (control) and dasatinib treatment groups. All mice were injected 100 μL of 10 μM CTX (Latoxan, Rhone-Alpes, France) into TA muscles under anesthesia. Dasatinib was dissolved in DMSO, diluted with sunflower oil, and injected intraperitoneally daily into the mice at 10 mg/kg/day. TA muscles were excised and snap-frozen in liquid nitrogen-chilled isopentane on days 0, 3, 7, 14, and 21 post-CTX injury. RXR inhibitor HX 531 (Cat#CAY20762, Cayman Chemical, Ann Arbor, MI, USA) was dissolved in DMSO, diluted with sunflower oil, and injected intraperitoneally daily into the mice at 7 µg/kg/day.

### Histology

For H&E or immunofluorescence staining, the freshly frozen muscle tissues were sectioned into 10 μm thick sections using a cryostat (CM1860, Leica Microsystems, Wetzlar, Germany). For immunofluorescence staining, sections were fixed with 4% paraformaldehyde (PFA) or ice-cold acetone for 5 min. Sections fixed with PFA were permeabilized with 0.1% Triton X-100 in PBS for 5 min and blocked with Blocking One (Nacalai Tesque Inc., Kyoto, Japan) for 5 min. The sections were incubated with the first antibodies at 4°C overnight, followed by incubation with the secondary antibodies for 1 h. Used antibodies are listed in **Supplemental Table 1**. Sections were mounted using SlowFade Diamond Antifade Reagent (Thermo Fisher Scientific, Waltham, MA, USA). Fluorescent images were captured using a confocal laser-scanning microscopy system A1Rsi (Nikon, Tokyo, Japan) or ECLIPSE Ti (Nikon, Tokyo, Japan). Images of the entire TA sections were captured to analyze the muscle fiber CSA and the number of the PDGFRα positive MSPs. The CSA, myofiber numbers, and the number of MSPs were quantified using WinROOF2021 (Mitani Corporation, Tokyo, Japan).

### Creatine kinase measurement

The blood samples collected from mice and centrifuged at 2000g for 20 min at 4°C to correct the serum. The serum level of creatine kinase was determined using the Mouse Creatine Kinase, Muscle/CKMM ELISA Kit (NBP2-75306, Novus Biologicals, Centennial, CO, USA). The absorbance was measured using a microplate reader iMark (Bio-Rad Laboratories, Hercules, CA, USA).

### Cell culture

The C2C12 myoblast cell line was purchased from the American Type Culture Collection (VA, USA). The cells were seeded on 96-well plates (167008, Thermo Fisher Scientific) or μ-Slide 8-well plates (ibidi, Gräfelfing, Germany) and cultured in a growth medium (GM; Dulbecco's modified Eagle's medium (DMEM) supplemented with 10% fetal bovine serum (FBS) and 1% penicillin-streptomycin). The cells were treated with dasatinib for 36 h, and cell viability was measured using a CCK-8 kit (Dojindo, Kumamoto, Japan). After cultivation till 90% confluency at 37°C in 5% CO_2_ and 20% O_2_, the cells were induced into myotubes using the differentiation medium (DM; DMEM supplemented with 5% horse serum and 1% penicillin-streptomycin). The cells were cultured at 37°C, 5% CO_2_, and 20% O_2_ for 2 days. The myotubes were fixed with 4% PFA for 5 min and permeabilized with 0.1% Triton X-100. Next, the myotubes were blocked and incubated with mouse anti- MyHC antibody (clone MF20, 1:2, DSHB) at 4°C overnight, followed by incubation with the Alexa Fluor 594-conjugated donkey anti-mouse IgG (1:1000, Jackson ImmunoResearch) antibody for 1 h. Images of myotubes were captured using a confocal laser scanning microscopy system A1Rsi (Nikon, Tokyo, Japan). MyHC-positive areas were analyzed using WinROOF2021 (Mitani Corporation, Tokyo, Japan).

### RNA extraction and real-time PCR analysis

Freshly frozen TA muscles were sliced to a thickness of 10 μm using a cryostat, and 100 sections were collected to extract RNA. Total RNA was extracted using a RNeasy Mini Kit (Qiagen, Hilden, Germany). The extracted RNA was reverse transcribed into cDNA using ReverTra Ace (TRT-101, TOYOBO, Osaka, Japan). Real-time PCR analysis was performed using the AriaMx Real-time PCR system (Agilent Technologies) with THUNDERBIRD® Next SYBR® qPCR Mix (QPX-201, TOYOBO). The PCR conditions were as follows: 95°C for 60 s, followed by 40 cycles of 95°C for 15 s, 60°C for 20 s, and 72°C for 30 s. Dissociation curve analysis was performed after amplification. The relative expression levels of the target genes were calculated using the ΔΔCt method. The *Cmas* gene was used as a reference gene to analyze target gene expression levels. The primers used are listed in **Supplemental Table 2**.

### Statistics

All statistical analyses were performed using Prism (version 9.5.1; GraphPad Software, San Diego, CA, USA). The equality of variance between the groups was analyzed using the F-test or Bartlett's test. Differences between the two groups were analyzed using a two-sided unpaired Student's t-test, and Welch's correction was applied when the variances between the groups were not equal. Differences between more than two groups were analyzed using a one-way analysis of variance (ANOVA), followed by Dunnett's post-hoc test when variances between the groups were not equal. Differences were considered statistically significant when the *p-*value was less than 0.05.

## Supplementary Material

Supplementary figure and tables.
