# Supplementary material for: Dasatinib promotes muscle differentiation and disrupts normal muscle regeneration
Source: Int J Med Sci. 2024 May 27;21(8):1461–71. doi: 10.7150/ijms.94938 (PMC11186431; doi:10.7150/ijms.94938)
Supplement: Supplementary file 1 — Supplementary figure and tables. [file ijmsv21p1461s1.pdf]

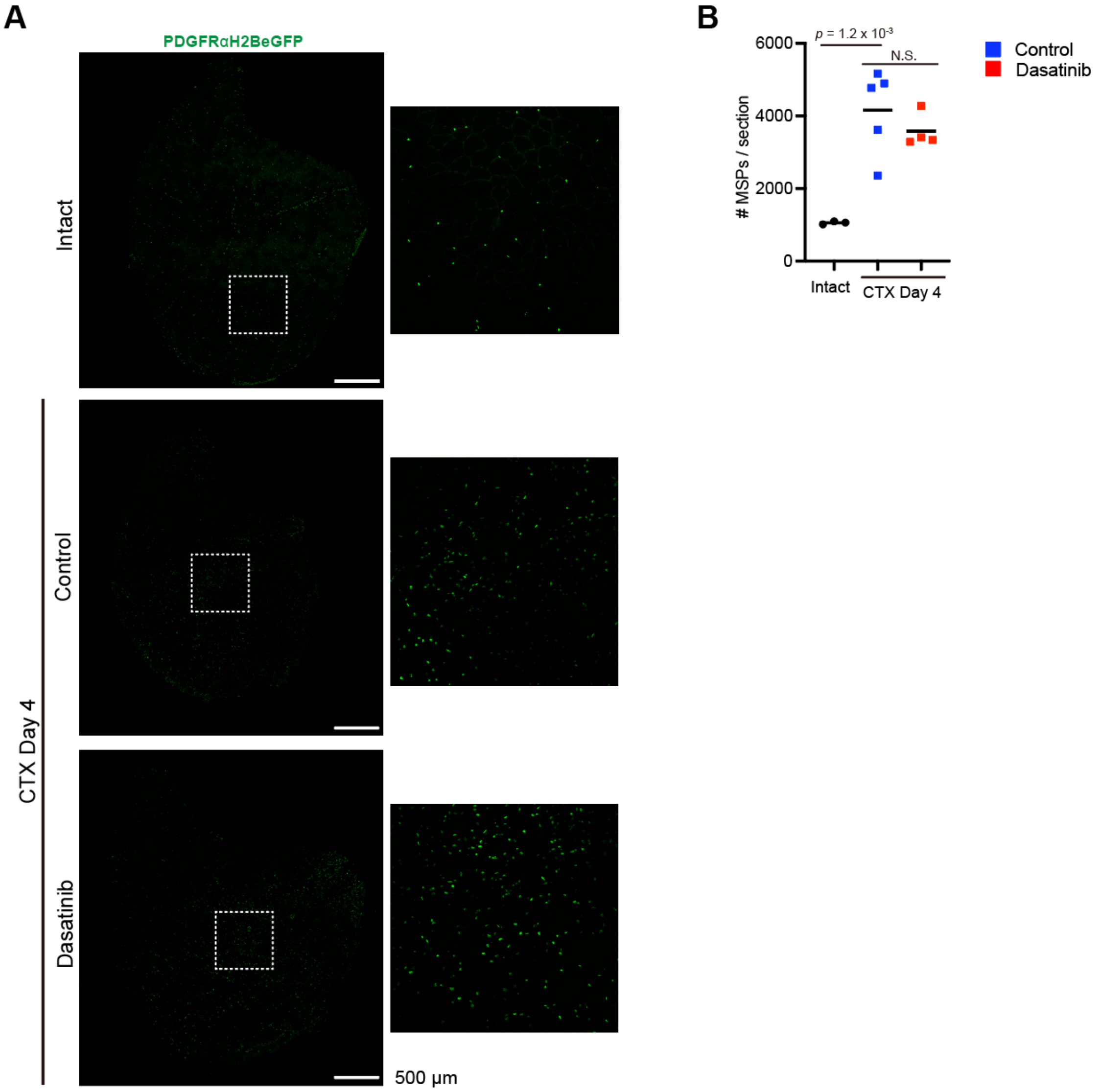

Supplemental Figure 1: Dasatinib does not suppress MSPs proliferation during muscle regeneration

The proliferation of MSPs identified the expression of platelet-derived growth factor receptor α (PDGFRα) was not prevented with dasatinib treatments at muscle regenerating states. **A.** Representative images of the TA cross-sections of PDGFRαH2BeGFP (*Pdgfra*^{EGFP}) mice at day 4 post-CTX injury are shown. The right panels show the magnified views of the boxed regions on the left panels. Scale bar = 500 µm. **B** Number of PDGFRα-positive MSPs in TA cross-sections of *Pdgfra*^{EGFP} mice at day 4 post-CTX injury. n = 3 for intact, n = 5 for no treatment (control) of CTX injury, and n = 4 for dasatinib treatment of CTX injury. Data represent individual data points and means. Data were analyzed using a one-way ANOVA test.

Supplemental Table 1. Antibodies used for IHC

**1st Antibody**	**Dilution**	**Supplier**
rat anti-laminin α2 antibody	1:200	Santa Cruz Biotechnology Cat.# sc-59854
anti-Myosin Heavy Chain	1:2	DSHB, clone: MF20
anti-MyHC I	1:4	DSMZ, Braunschweig, Germany, clone: BA-F8
anti-MyHC IIA	1:4	DSMZ, Braunschweig, Germany, clone: SC-71
anti-MyHC IIB	1:4	DSMZ, Braunschweig, Germany, clone: BF-F3
rabbit anti-laminin	1:400	Sigma-Aldrich, St. Louis, MO, USA, #L9393

**2nd Antibody**	**Dilution**	**Supplier**
Alexa 594®-conjugated anti rat IgG	1:1000	Thermo Fisher Scientific, Waltham, MA, USA
Alexa 594®-conjugated anti mouse IgG	1:1000	Thermo Fisher Scientific
Dylight 405 anti-mouse IgG2b	1:1000	Jackson Immuno Research, West Grove, PA, USA, #115-475-207
Alexa Fluor 555 anti-mouse IgG1	1:1000	Thermo Fisher Scientific, Waltham, MA, USA, #A21127
Alexa Fluor 647 anti-mouse IgM	1:1000	Thermo Fisher Scientific, #A21238
Alexa Fluor 488 antirabbit IgG	1:1000	Thermo Fisher Scientific, #A32790
DAPI	1:5000	DOJINDO

Supplemental Table 2. Primers used in qRT-PCR

**Gene**	**Direction**	**Sequence**
Cmas	Forward	5’-CAAAGGCATCCCACTGAAGA-3’
	Reverse	5’-CCCACACACTCTGGAAGACC-3’
Pdgfra	Forward	5’-TCAGCTGTCTCCTCACAGGG-3’
	Reverse	5’-ACTCTCCCCAACGCATCTCA-3’
Myod1	Forward	5’-GGCAGAATGGCTACGACACC-3’
	Reverse	5’-AGATGCGCTCCACTATGCTG-3’
Myogenin	Forward	5’-CAGTACATTGAGCGCCTACAG-3’
	Reverse	5’-GGACCGAACTCCAGTGCAT-3’
Myoz1	Forward	5’-GGAACTTGGCATTGACCTACTG-3’
	Reverse	5’-AAACTTGGGCATCTGGAAGG-3’
Mymx	Forward	5’-CTGAGCTGTCTGCTCTTTGT-3’
	Reverse	5’-TCTCCTTCCTCTGGGAGTG-3’
Mymk	Forward	5’-ATCGCTACCAAGAGGCGTT-3’
	Reverse	5’-CACAGCACAGACAAACCAGG-3’
Dmd	Forward	5’-AAGAGGAAGAAATGCCCCCG-3’
	Reverse	5’-CCATGCGGGAATCAGGAGTT-3’
Myh1	Forward	5’-GGAGGACCAAGTGAGTGAGC-3’
	Reverse	5’-TCGTCTAGCTGGCGTGAGTA-3’
Myh2	Forward	5’-AGCGACTGATCAACGACCTG-3’
	Reverse	5’-AACTGAGATACCAGCGCTTCC-3’
Myh4	Forward	5’-AAACCACCTCAGAGTTGTGGA-3’
	Reverse	5’-GTTCCGAAGGTTCCTGATTGC-3’
Myh7	Forward	5’-GCCCTTTGACCTCAAGAAAG-3’
	Reverse	5’-CTTCACAGTCACCGTCTTG-3’
